# Development and validation of a Context-sensitive Positive Health Questionnaire (CPHQ): A factor analysis and multivariate regression study

**DOI:** 10.1186/s41687-024-00718-8

**Published:** 2024-04-12

**Authors:** Brian M. Doornenbal, Tim van Zutphen, Lise F. E. Beumeler, Rimke C. Vos, Mark Derks, Hinke Haisma, M. Elske van den Akker-van Marle, Jessica C. Kiefte-de Jong

**Affiliations:** 1 Department of Public Health and Primary Care/Health Campus The Hague, Leiden University Medical Center, Albinusdreef 2, Leiden, 2333 ZA the Netherlands; 2 Salut Holding B.V., Arnhem, the Netherlands; 3 Campus Fryslân, University of Groningen, Leeuwarden, the Netherlands; 4 Biomedical Data Sciences, Leiden University Medical Center, Leiden, the Netherlands; 5 Research Center Positive Health, Lifestyle, and Leadership, Saxion University of Applied Sciences, Enschede, the Netherlands

**Keywords:** Positive Health, Capability Approach, Concurrent validity, Factor analysis, Factorial validity, Public health, Primary care

## Abstract

**Background:**

The concept of Positive Health (PH) has gained increasing attention as a way of measuring individuals’ ability to adapt in the face of contextual challenges. However, a suitable measurement instrument for PH that encompasses contextual factors has not yet been developed. This paper responds to this need by developing a Context-specific Positive Health (CPH) measurement instrument that aligns with the Capability Approach (CA).

**Methods:**

The measurement instrument was developed and tested among a representative sample of 1002 Dutch internet survey panel members with diverse sociodemographic backgrounds. The instrument was developed in two stages: a preparation phase consisting of focus groups and expert consultations, and a validation among a representative panel of Dutch citizens. The goal of the preparation phase, was to pilot test and refine previously proposed Positive Health questionnaires into an initial version of the CPHQ. The validation phase aimed to examine the initial CPHQ’s factorial validity using Factor Analysis, and its concurrent validity using Multivariate Regression Analysis.

**Results:**

The developed questionnaire demonstrated adequate factorial and concurrent validity. Furthermore, it explicitly includes an assessment of resilience, this being a key component of PH.

**Conclusions:**

The introduced measurement tool, the CPHQ, comprises 11 dimensions that we have labeled as follows: relaxation, autonomy, fitness, perceived environmental safety, exclusion, social support, financial resources, political representation, health literacy, resilience, and enjoyment. In this article, we present four major contributions. Firstly, we embedded the measurement in a theoretical framework. Secondly, we focused the questionnaire on a key concept of Positive Health - the “ability to adapt.” Thirdly, we addressed issues of health inequality by considering contextual factors. Finally, we facilitated the development of more understandable measurement items.

**Supplementary Information:**

The online version contains supplementary material available at 10.1186/s41687-024-00718-8.

## Background

Although institutions and organizations frequently introduce policies and practices aimed at improving health, it is often unclear how to define and measure health [1–3]. Recent policies and interventions increasingly refer to Positive Health (PH)– that is, “the ability to adapt and self-manage in the face of social, physical, and emotional challenges” [4, 5]. However, a measurement instrument for PH, which is needed for monitoring and evaluation purposes, has not yet been fully developed [6–8]. A questionnaire-based PH dialogue tool exists, but this tool aims to inspire conversations about health during the consultation of an individual with their health professional instead of measuring health [7, 9]. Therefore, it is crucial to further develop and validate a suitable measurement instrument to measure health in line with the concept of PH.

To develop a measurement instrument for PH, scholars previously examined the suitability of the PH dialogue tool. Based on tests of factorial validity, the 42 items of the PH dialogue tool were turned into a 17-item model (PH-17), that comprised six factors: physical fitness, mental functions, future perspective, contentment, social relations, and daily life-management [8]. Philippens and colleagues [10] provided support for the construct validity of this measurement model by finding a positive impact of a combined lifestyle intervention on PH-17. Subsequent tests of concurrent validity showed that PH-17 explained over 50% of the variance in measurements of self-rated health and happiness, but less than 25% of the variance in measurements of autonomy, personal growth, stability, and self-care [6]. For institutions and organizations to use a PH measurement, scholars should resolve concerns about the fit between the measurement of PH and what this measurement purports to measure [6, 7]. PH is denoted as “the ability to adapt and self-manage in the face of social, physical, and emotional challenges” [4, 5], but the PH-17 measurement model does not clearly encompass a measurement of contextual challenges that persons may face [6]. Contextual factors, such as neighborhood adversity, perceptions of discrimination, and social resources, can hamper coping or recovery processes [11].. Therefore, a measurement of the ability to adapt should take these contextual factors into consideration.

**Fig. 1:**
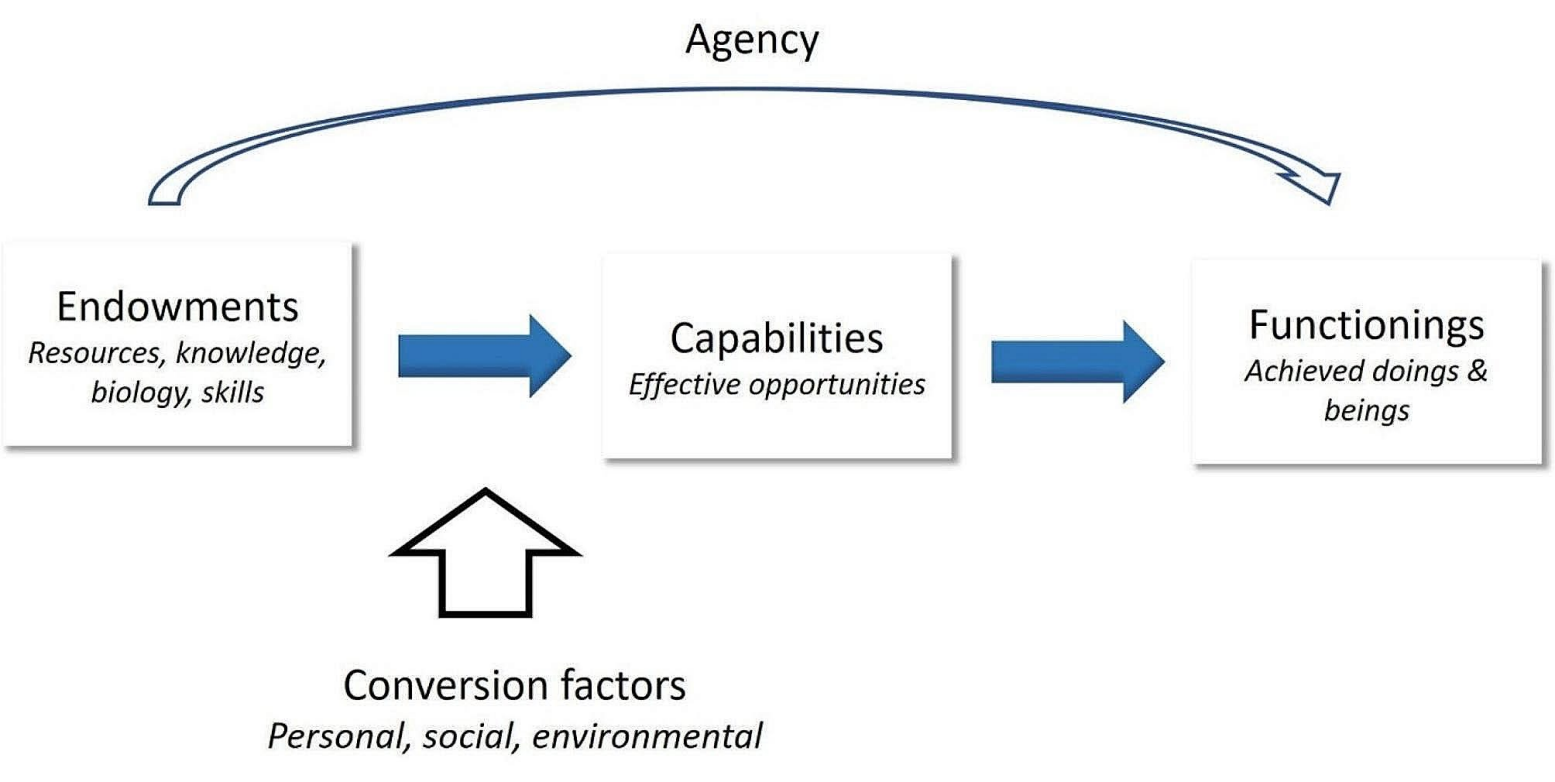
Conceptual framework of the Capability Approach: Mapping the transformation from endowments to functionings with agency as a mediator and conversion factors as moderator

To account for these contextual factors and to advance the measurement of PH, scholars can learn from the Capability Approach (CA) framework (see Fig. 1). CA claims that well-being should be understood in terms of so-called functionings and capabilities. Functionings are people’s valued doings and beings, such as being educated and being well-nourished. Capabilities are the opportunities that people can choose from to achieve these valued outcomes. Person’s capabilities depend on resources and on the contextual factors to make use of these resources, such as personal, social, and environmental conditions, which are referred to in the CA literature as conversion factors [12–14]. As a result of inequality in conversion factors, such as a personal background or social network that limits opportunities, people can have different (health) capabilities. This approach is different from a utilitarian approach where it is assumed that the availability of resources will result in improved outcome measures. For example, in a utilitarian approach appropriate knowledge and availability of healthy foods would result in providing healthy meals. However, when applying a Capability Approach, we would also consider contextual aspects. If knowledge and healthy foods would be in place but external factors, such as unemployment of the parents or children dropping out of school, i.e. a household environment that would favor harmony at the dinner table over making healthy choices [15], the capability to provide a healthy meal may not be guaranteed and health outcomes could be deprived. In the Capability Approach, external factors are referred to as conversion factors. Such an approach calls for different cues to action, and can improve inequalities, in our case health inequalities.

In this paper, we respond to the need for a valid measurement of PH by advancing the previously developed PH-17 measurement into a measurement that takes into account contextual factors recognizing that individuals’ capabilities related to health can be influenced by a variety of social determinants, and that policies and interventions aimed at reducing health inequalities should address these underlying factors. By taking into account contextual factors, we aim to align the measurement of PH more with the definition of PH– that is, “the ability to adapt and self-manage in the face of social, physical, and emotional challenges”. We will test the factorial validity and concurrent validity of the advanced measurement that we will develop, which we denote in this paper as Context-sensitive Positive Health (CPH), similar to previous efforts [6, 8]. Beyond this empirical approach, we take a theoretical approach by learning from the CA [12–14]. 

This instrument is not only designed for monitoring and evaluation purposes but has the potential to serve as a crucial tool for directing health policy. The CPHQ can be used to identify domains where specific groups—such as employees within an organization or residents in a community—are deprived and may benefit from targeted health interventions that would address context. By enabling the identification of areas with potential for improvement, the CPHQ can inform policymakers and healthcare providers about where to allocate resources and efforts to enhance overall health outcomes.

In this article, we present four major contributions. First, it helps to embed the construct of CPH in a theoretical framework - which is needed for theory building and testing [7]. Second, it creates clarity about the focus of CPH - that is, on the “ability to adapt” and the enabling dimensions. Third, it accommodates for issues of health equity that heavily depend on the conditions in which people are born, grow, live and age in [11]. Fourth, it provides a structure for creating more comprehensible measurement items - which scholars called for [7]. 

## Methods

The Context-sensitive Positive Health Questionnaire (CPHQ) was developed in two phases: a preparation phase consisting of focus groups and expert consultations and a validation in a representative panel of citizens. As input for these phases, a questionnaire was used. The preparation phase was intended to pilot test and refine this questionnaire with both public health experts (e.g., specialized in poverty) and citizens from different backgrounds (e.g., educational, cultural, health conditions). The goal of the validation phase was to examine the factorial validity and concurrent validity of the refined questionnaire.

The questionnaire that served as a starting point was inspired by the Positive Health dialogue tool [5]. The Positive Health dialogue tool consists of 42 statements, each belonging to one of six dimensions, initially named: bodily functions, mental functions and perception, spiritual existential dimension, quality of life, social and societal participation, and daily functioning [8]. The 42 PH statements were extended with items related to Nussbaum’s 10 core capabilities for adult-wellbeing [16], focusing on the context of individuals. We developed the context items on existing validated quality of life questionnaires and other context-sensitive sources such as the resilience monitor, self-sufficiency matrix, monitor broad welfare, and livability index [17]. These items were divided into interpersonal context (16 items), social context (24 items), and environmental context (14 items). Specific contexts can function as conversion factors that either support or hinder people’s capabilities [14]. Examples of conversion factors are gender, ethnicity, culture, laws and regulations or characteristics of the physical environment [14]. The initial questionnaire items, including the PH-items, were formulated in line with the Capability Approach [12, 16]. We rephrased items such that they focused on endowments, capabilities, or functionings rather than states or outcomes. For example, “I know what I can and what I can’t” was rephrased as “I am able to perform tasks and activities adequately.” Based on the focus groups and expert input, the questionnaire was extended with items that were considered to be missing by the citizens and/or experts which were related to resilience, social support, relaxation, and autonomy.

### Preparation phase: focus groups

The preparation phase started with focus groups. The focus groups were aimed to assess the relevance and comprehensibility of the 42 PH-items as well as the contextual items about perceived health. We included participants distributed over various age groups, genders, health conditions (with or without chronic disease), socioeconomic background, and cultural backgrounds in Northern and Southern parts of the Netherlands via existing primary and social care networks as well as local informal networks of citizen initiatives. Our recruitment strategy was designed to ensure a diverse and inclusive representation of participants. This included reaching out to potential participants through general practitioners in socioeconomically disadvantaged areas, aiming to capture a wide array of perspectives across different demographic characteristics. In total, we organized six focus group sessions, each with five participants, which appeared to be sufficient to achieve saturation. Two focus groups were organized online due to COVID-restrictions; one with members from rural communities in the Northern Netherlands and another involved experts by experience in poverty.

As a preparation for the focus group participants were asked to fill in the 42 PH-items. During the focus groups of ± 1.5-2 h, the participants were first asked how they defined perceived health in their own words. Next, all domains of the 42 PH-items conversation tool were discussed if they were relevant in light of their definition of perceived health, the comprehensibility, and whether items were missing. Because resilience was previously mentioned as a core element in the original definition of Huber who proposes to see health more as a power than a state, defined as the power to be resilient [4] and previous research showed that the initial PH scales explained little variation in resilience [6], we specifically asked citizens whether resilience was sufficiently reflected and whether it would be of added value to add one or more other ‘potential’ items to the PH model. For the contextual items, participants were asked to describe facilitators and barriers for health maintenance as well as health promotion in their personal lives and whether or not these barriers relate to each of the ten core capabilities of Nussbaum. At least two moderators led the discussion and encouraged everyone to participate while one of the researchers of the team observed and made field notes while paying attention to the time schedule. All online focus groups were audiotaped and transcribed verbatim. For the PH items the team started with open coding, followed by axial coding and the last step was selective coding according to the main themes of the PH model and additional items that were missed by the citizens in the initial PH model and further analyzed according to the endowments, capabilities, and conversion factors from the Capability Approach. Contextual items were derived from the conversion factors. Coding was performed by 3 members of the research team followed by the qualitative analysis of the codes.

### Preparation phase: expert consultations

After the focus groups, expert consultations took place. Experts from the medical, policy, and research domains, as well as poverty experience were consulted from the researcher’s academic network as well as the network of the Dutch Institute of Positive Health. Through an anonymous questionnaire, the experts were asked to mention stronger and weaker points of the definition of health and PH and the domains and items belonging to this definition. After that, they could indicate and rank the most important domains of health from a list with domains from different questionnaires, such as PH-42 [8], EQ-5D [18], ICECAP-A [19], and HR-SWB [20]. Also, new domains could be listed. Subsequently, the individual items of the PH were shown for which respondents could indicate which of them belong to health. The goal of the expert consultation was to further refine the questionnaire before empirically testing the factorial validity and concurrent validity.

In total, 20 experts were consulted individually on the items about PH as well as the personal, social and environmental context. They were asked for confirmation on relevance of the items and on possible missing contextual items to add to the 42-item PH questionnaire. The experts that participated were almost exclusively from Dutch universities with expertise in social sciences, medical sciences, cultural geography, population health, governance and economics.

In addition, items were refined. For example, poverty experience experts indicated that they were hesitant to complete the questionnaire because the formulation of some items did not align with their reality. Based on this feedback, the language was adjusted. For example, positively phrased questions such as “I do not have financial problems” were rephrased as “I have debts” to better reflect a low socio-economic situation. In total, the 42 items were complemented with 12 items on autonomy, relaxation, resilience, and social support to a total of 54 items developed and formulated based on the Capability Approach and complemented with 16 items on personal context, 24 items on social context and 14 items on environmental context (See Supplemental Table 1).

### Validation in a representative internet survey panel of Dutch citizens

The goal of the validation phase was to examine the factorial validity and concurrent validity of the refined CPHQ questionnaire. The data used in these analyses were collected through Flycatcher, a Dutch independent Internet panel and a spin-off from Maastricht University. Operating in accordance with ISO standards, this panel ensures high-quality and reliable data collection processes. Recruitment for the study was facilitated by Flycatcher, utilizing their panel of over 10,000 individuals aged 18 and older. Panel members have voluntarily joined through a ‘double-active-opt-in’ process, guaranteeing their active and informed consent for participation in online surveys. As compensation, panel members are rewarded for each completed questionnaire with a points system, where accumulated points can be redeemed for gift certificates. This internet panel is purposefully designed to be representative of the Dutch population. For our study, a sample of 1632 was randomly selected from the original panel of which 1002 panel members participated (response 61.5%). In terms of demographic variables, the internet panel members participating in this study (e.g., 50.0% women; education levels: low = 28.3%, medium = 43.8%, high = 27.8%) were representative of the general Dutch population (See supplementary Table 4; 50.3% women; education levels: low = 28.3%, medium = 37.7%, high = 34.0%) The data collection occurred in December 2020, involving a structured approach wherein participants were invited via email to complete an online survey.

The CPHQ was compared with other health scales to assess the concurrent validity—that is, the degree to which a new test compares to an established test. For this, participants were asked to fill out the adapted CPHQ, consisting of 54 items. Each item in the CPHQ was rated on a 5-point Likert scale, where 1 represented “strongly disagree” and 5 represented “strongly agree”. Participants. In addition to the adapted CPHQ, participants were also asked to fill out the following validation scales.

### Brief resilience scale (BRS)

To measure resilience, the three positive-worded items of the Brief Resilience Scale (BRS) were used as items of Positive Health are also worded positively. The original Brief Resilience Scale (BRS), generated by Smith et al. [21] has a good internal consistency with Cronbach’s α ranging from 0.81 to 0.91 and a one factor structure. The items that were used for the present study were: (i) “I tend to bounce back quickly after hard times”, (ii) “It does not take me long to recover from a stressful event”, and (iii) “I usually come through difficult times with little trouble”. Answers were given on a 5-point Likert scale ranging from “*strongly disagree*” (= 1) to “*strongly agree*” (= 5).

### Health-related subjective well-being (HR-SWB)

Health-related subjective well-being (HR-SWB) was measured using the measurement proposed by De Vries and colleagues [20]. This measurement comprises five dimensions: (i) bodily independence, (ii) happiness, (iii) loneliness, (iv) autonomy, and (v) personal growth. Each domain was measured using one item, such as “I feel lonely” (loneliness). Previous work by de Vries et al. showed adequate factorial validity with five factors explained 65% of the total variance in a Dutch Population. They were, however, not tested on validity and reliability [20]. Responses were rated on a 5-point Likert scale ranging from “*strongly disagree*” (= 1) to “*strongly agree*” (= 5).

### EuroQol five-dimensions (EQ-5D)

The EQ-5D-5 L (EuroQol five-Dimensions) captures the following domains of health: mobility, self-care, usual activities, pain/discomfort, and anxiety/depression. Answer categories of questions on these were assessed on a 5-point Likert scale. At last, a visual analog scale was used to measure the overall self-rated health of the respondent that day. The EQ-5D-5 L has been tested in The Netherlands showing moderate to good test-retest-reliability (ICC = 0.81 for the total score and 0.64 for the visual analog scale) [18].

### ICEpop CAPability measure for adults (ICECAP-A)

The ICECAP-A (ICEpop CAPability measure for Adults) was used to measure well-being following the Capability Approach in terms of individuals’ capabilities [19]. The measurement comprises five domains: stability, attachment, autonomy, achievement, and enjoyment. Each of these domains was measured using one statement on a 4-point scale. The ICECAP-A has been tested in The Netherlands and showed adequate construct validity and good test-retest-reliability (ICC = 0.79).

### Analyses

An Exploratory Factor Analysis (EFA), followed by a Confirmatory Factor Analysis (CFA) was conducted to test the factorial validity—that is, the extent to which a putative structure of a scale is recoverable in a set of test scores. As we had no a priori hypotheses regarding how the newly developed items, inspired by the Capability Approach, would align with the Positive Health framework, we derived a factor structure using EFA. This structure was then tested using CFA. For the analyses, the data (*n* = 1002) were randomly and evenly partitioned into two datasets: a training and a test dataset. The training dataset was used for the Exploratory Factor analysis (EFA), whereas the test dataset was used for the CFA. Partitioning the data into a training and test dataset helps to evaluate how well unknown data fit the measurement model.

To examine the dimensionality of the data, a series of factor models were fitted. We began with a one-factor model and incrementally added one factor (k + 1) at a time. While fitting the models, all items were allowed to load on all the factors on the model– no a priori restrictions were imposed on the factorial structure. Factors were added to the model until the model (i) demonstrated adequate goodness of fit in terms of the Comparative Fit Index (CFI) and Tucker-Lewis Index (TLI), the Root Mean Square Error of Approximation (RMSEA), and the Standardized Root Mean Square Residual (SRMR); (ii) explained most of the variance; (iii) had an interpretable structure in which at least two items load strongly (i.e., ≥ 0.40) on each factor only (i.e., no cross-loadings ≥ 0.40 allowed).

The goodness of fit was assessed using CFA with robust Maximum Likelihood (MLR). Compared to Maximum Likelihood (ML) estimation, MLR is less dependent on the assumption of multivariate normal distribution [22]. To compute the goodness of fit indices (i.e., CFI, TLI, RMSEA, and SRMSR), items were selected during the EFA. The three items with the highest factor loadings (≥ 0.40) and without (≥ 0.40) cross-loadings were selected to compute the goodness of fit indices.

While exploring the factor structure, Horn’s parallel analysis was applied [23, 24] to limit our search to dimensionalities for which the likelihood is greater than random chance. Specifically, the *k*th eigenvalue of the sample covariance was compared with the sampling distribution of the *k*th eigenvalue obtained through Monte Carlo simulation from random independent data. Only the factor structures for which the *k*th eigenvalue of the sample data is substantially larger than the *k*th eigenvalue of the simulated data have a dimensionality that is greater than one would expect by random chance.

To evaluate the relationship between CPHQ and other measurements of health (i.e., BRS, HR-SWB, EQ-5D, ICECAP-A), multivariate regression analyses were conducted. During each regression analysis, the factors of the final measurement model of CPHQ were used as independent variables, whereas the other measurements of health (incl. underlying domains) were each time used as a dependent variable. These were not multivariate regression analyses with multiple dependent variables assessed simultaneously; instead, each model focused on a single dependent health outcome variable, with the intention to assess its relationship with the multidimensional CPHQ construct. The proportion of the variance for the dependent variable that is explained by the independent variables in the regression models (R^2^) was used as a statistical measure that represents the strength of the statistical relationships between CPHQ and the other health domains—which we denote as validation scales. This analysis was intended to assess the concurrent validity.

We hypothesize significant R² values for the multivariate relationship between the CPHQ and the validation scales, reflecting our anticipation that the CPHQ contributes to health measurement, a function also served by the validation scales. However, very high R² values might suggest statistical redundancy between the CPHQ and existing measures. Additionally, we hypothesize that each dimension of the CPHQ must demonstrate a significant relationship with at least one of the validation scales. This is essential to confirm that all dimensions are statistically relevant for commonly used health measurements.

#### Hypothesis 1

We expect that there will be significant multiple correlation coefficients (R² values) for the multivariate relationship between the CPHQ and the validation scales.

#### Hypothesis 2

We hypothesize that each dimension of the CPHQ demonstrates a significant relationship with at least one of the validation scales.

The tests of factorial validity (factor analysis) and concurrent validity (regression analyses) were conducted using R statistical software, version 4.3.2.

## Results

Inspection of data suggested that the training dataset was suitable for EFA. The adequacy of the sample size for the EFA (*n* = 501) was “very good” [25], with a subject-to-item ratio of 4.5:1. The Kaiser-Meyer-Olkin (KMO) test yielded a statistic of 0.95, suggesting the data set contains a significant proportion of variance among variables that might be common variance (caused by underlying factors). Bartlett’s test of sphericity yielded significant results, χ2(111) = 3095.04, p < 0.001, implying that the data are suitable for performing factor analysis because the correlations among variables are greater than one would expect by chance.

Using the training data to explore the factor structure, we limited the search to 15 factors because Horn’s parallel analysis [23, 24] suggested that a dimensionality of more than 15 factors is unlikely compared to the dimensionality expected by random chance. This search was further narrowed down to 11 dimensions, because for 12 dimensions and more, at least one dimension did not have strong item loadings (i.e., exceeding 0.40). As reported in Supplemental Table 2, most variance (> 50%) was explained when the data were structured into 9 dimensions or more. Thus, we focused on factor structures ranging from 9 to 11 dimensions.

During the subsequent CFA, the items showed positive factor loadings on the respective domains with an average standardized coefficient of 0.793, ranging from 0.543 to 0.900 (Table 1). Thus, an 11 dimensions factor model adequately described the data. Across the factor structures ranging from 9 to 11 dimensions, an 11-dimension factor structure had the best goodness of fit indices (CFI = 0.944, TLI = 0.932, RMSEA = 0.049, SRMR = 0.050). The goodness of fit indices were computed based on the test data (*n* = 501)– i.e., the data that were not used during the EFA. The 11 dimensions solution had an interpretable factor structure in which at least two items load strongly (i.e., ≥ 0.40) on each factor only during the EFA (Table 2).

**Table 1:** Standardized weights of the exploratory eleven-factor model using oblimin rotated factors

Domain	Item	F1	F2	F3	F4	F5	F6	F7	F8	F9	F10	F11
Context	I am able to relax when necessary.	**0.835**	-0.030	0.029	0.054	-0.029	0.005	0.073	0.035	-0.035	0.061	-0.071
Context	I have enough peace of mind.	**0.776**	-0.017	0.004	0.046	-0.061	-0.012	0.013	0.035	-0.009	0.118	0.044
PH	I am able to unwind.	**0.710**	0.02	0.094	-0.022	0.094	0.095	-0.002	0.028	0.011	-0.09	0.043
PH	I am capable of carrying out tasks and activities adequately.	0.032	**0.697**	0.103	0.031	0	-0.009	0.072	-0.034	0.048	0.075	0.067
PH	I am able to participate in activities that I value in my daily life (work, study, etc.).	0.073	**0.671**	0.095	-0.03	0.007	0.062	0.139	0.054	-0.067	0.072	0.05
PH	I can work/volunteer.	-0.002	**0.657**	0.193	-0.022	-0.024	0.081	0.055	0.09	-0.088	0.034	-0.049
PH	I feel healthy.	0.059	0.023	**0.802**	0.014	-0.006	0.018	-0.016	-0.03	0.023	0.027	0.089
PH	I feel in good health.	0.092	-0.002	**0.763**	0.053	-0.054	0.068	0	-0.056	-0.064	0.042	0.106
PH	I can move easily, such as climbing stairs, walking, or cycling.	-0.056	0.141	**0.763**	0.002	0.011	-0.045	0.036	0.057	0.014	-0.041	-0.034
Context	I feel safe in the neighborhood where I now live.	-0.052	0.004	0.04	**0.724**	0.136	0.015	0.05	0.059	0.039	0.04	-0.003
Context	My home environment is safe and provides numerous opportunities to engage in daily life.	0.046	0.073	0.052	**0.663**	0.024	-0.018	0.114	0.037	0.017	-0.056	-0.022
Context	I feel connected to the environment where I now live.	0.123	-0.032	-0.043	**0.576**	-0.079	0.167	-0.024	0.069	-0.028	0.061	-0.015
Context	I feel disadvantaged because of my religion or spiritual beliefs.	-0.013	0.024	0.003	0.019	**0.847**	0	-0.019	0.041	0.03	-0.025	-0.026
Context	I feel disadvantaged because of my (cultural) background.	0.029	0.023	-0.023	0.04	**0.843**	-0.024	0.033	0.06	0.015	-0.055	0.012
Context	I feel disadvantaged or excluded based on my sexuality and/or gender.	-0.03	0.065	-0.05	0.075	**0.728**	-0.029	0.026	-0.003	0.005	0.054	0.04
PH	I can find people with whom I can have a good time.	0.06	0.02	0.144	0.027	0.076	**0.617**	0.094	0.011	0.036	-0.056	0.044
PH	I feel that people support me when needed.	0.084	-0.01	0.065	0.114	0.015	**0.607**	0.098	0.025	0.114	-0.085	-0.014
PH	I feel that I ‘fit in’ in my environment.	0.09	0.056	-0.1	0.065	0.056	**0.587**	0.07	-0.024	-0.04	0.11	0.154
Context	I can afford to eat healthily and participate in physical activities.	0.031	-0.004	0.06	0.025	-0.019	-0.008	**0.863**	0.005	0.073	-0.02	-0.028
Context	I have enough money to do things that are important to me.	0.04	0.015	-0.014	-0.027	-0.024	-0.016	**0.842**	0.077	-0.042	0.039	0.014
PH	I can afford to live a healthy lifestyle.	-0.048	0.084	0.006	0.122	-0.001	0	**0.775**	-0.021	0.018	-0.037	0.076
Context	Politics makes me feel represented.	0.025	0.012	-0.033	-0.004	-0.01	-0.069	0.005	**0.898**	0.01	0.047	0.024
Context	I feel confident in the way that politicians handle issues that are important to me.	0.003	-0.026	0.015	0.016	0.097	0.011	-0.014	**0.864**	0.005	-0.06	-0.02
Context	I can communicate with healthcare professionals and understand their explanations of my illness or treatment.	0.018	-0.047	-0.051	0.011	-0.019	0.055	0.087	0.125	**0.65**	0.057	-0.078
Context	I know where to go for medical assistance.	0.015	0.016	-0.045	0.214	0.144	0.041	0.04	-0.1	**0.511**	0.009	0.04
Context	When I look up or receive information about a subject, it is explained in a way that I can understand.	-0.069	0.059	0.055	0.024	0.04	0.015	0.019	0.093	**0.482**	0.005	-0.016
BRS	When something bad happens, it is difficult for me to move on.	-0.005	-0.018	0.033	0.043	0.004	0.003	0.032	-0.023	0.077	**0.69**	-0.044
BRS	I have a hard time getting through stressful situations.	0.086	0.021	-0.012	-0.009	0.079	-0.011	-0.012	0.007	0.023	**0.64**	0.03
BRS	I don’t need much time to recover from a stressful event.	0.20	0.066	-0.012	0.024	-0.119	-0.061	0.076	0.032	0.1	**0.437**	0.034
PH	I feel happy.	0.126	-0.085	0.122	0.074	0.02	0.102	-0.015	0.068	-0.059	0.063	**0.661**
PH	I am able to enjoy life.	0.098	0.073	0.031	0.036	0.05	0.084	0.072	0.052	0.002	-0.049	**0.646**
PH	I am able to be grateful for what life has to offer.	-0.018	0.028	0.049	0.063	0.099	0.029	0.067	0.04	0.037	-0.04	**0.622**

*Note.* F1 = Relaxation, F2 = Autonomy, F3 = Fitness, F4 = Perceived environmental safety, F5 = Exclusion, F6 = Social support, F7 = Financial resources, F8 = Political representation, F9 = Health literacy, F10 = Resilience, F11 = Enjoyment, PH = Positive Health, BRS = Brief Resilience Scale

**Table 2:** Parameter estimates Confirmatory Factor Analysis (CFA) using robust Maximum Likelihood (MLR)

Latent Factor	Description	B	SE	Z	β	p
F1	I am able to relax when necessary.	1.000			0.894	
F1	I have enough peace of mind.	1.046	0.045	23.254	0.817	***
F1	I am able to unwind.	0.785	0.042	18.670	0.788	***
F2	I am capable of carrying out tasks and activities adequately.	1.000			0.785	
F2	I am able to participate in activities that I value in my daily life (work, study, etc.).	1.351	0.096	14.090	0.881	***
F2	I can work/volunteer.	1.292	0.116	11.128	0.689	***
F3	I feel healthy.	1.000			0.890	
F3	I feel in good health.	1.075	0.043	25.022	0.873	***
F3	I can move easily, such as climbing stairs, walking, or cycling.	0.899	0.064	14.152	0.660	***
F4	I feel safe in the neighborhood where I now live.	1.000			0.845	
F4	My home environment is safe and provides numerous opportunities to engage in daily life.	0.725	0.080	9.089	0.644	***
F4	I feel connected to the environment where I now live.	0.916	0.102	8.976	0.582	***
F5	I feel disadvantaged because of my religion or spiritual beliefs.	1.000			0.870	
F5	I feel disadvantaged because of my (cultural) background.	1.009	0.051	19.755	0.890	***
F5	I feel disadvantaged or excluded based on my sexuality and/or gender.	0.900	0.061	14.795	0.738	***
F6	I can find people with whom I can have a good time.	1.000			0.753	
F6	I feel that people support me when needed.	0.965	0.078	12.368	0.803	***
F6	I feel that I ‘fit in’ in my environment.	1.101	0.073	14.995	0.814	***
F7	I can afford to eat healthily and participate in physical activities.	1.000			0.881	
F7	I have enough money to do things that are important to me.	1.155	0.052	22.115	0.854	***
F7	I can afford to live a healthy lifestyle.	0.889	0.057	15.580	0.805	***
F8	Politics makes me feel represented.	1.000			0.888	
F8	I feel confident in the way that politicians handle issues that are important to me.	1.014	0.099	10.199	0.900	***
F9	I can communicate with healthcare professionals and understand their explanations of my illness or treatment.	1.000			0.812	
F9	I know where to go for medical assistance.	0.887	0.078	11.342	0.776	***
F9	When I look up or receive information about a subject, it is explained in a way that I can understand.	0.682	0.081	8.444	0.543	***
F10	When something bad happens, it is difficult for me to move on.	1.000			0.829	
F10	I have a hard time getting through stressful situations.	1.008	0.054	18.804	0.782	***
F10	I don’t need much time to recover from a stressful event.	0.713	0.078	9.179	0.614	***
F11	I feel happy.	1.000			0.880	
F11	I am able to enjoy life.	0.918	0.039	23.694	0.888	***
F11	I am able to be grateful for what life has to offer.	0.679	0.051	13.225	0.696	***

*Note.* *** =p < 0.001; B = unstandardized estimates; SE = standardized error; F1 = Relaxation, F2 = Autonomy, F3 = Fitness, F4 = Perceived environmental safety, F5 = Exclusion, F6 = Social support, F7 = Financial resources, F8 = Political representation, F9 = Health literacy, F10 = Resilience, F11 = Enjoyment

Furthermore, to provide a comprehensive understanding of the item-level responses, we have included detailed item descriptives in a Supplemental Table 3. This table presents the frequency distribution of responses for each item on the 5-point Likert scale. Notably, the Autonomy domain (F2) showed that a majority of 85% of respondents felt self-sufficient in managing tasks, reflecting a strong perception of individual agency. Similarly, in the Perceived environmental safety domain (F4), 83% of participants agreed that they feel secure in their living environments. The most striking distribution is observed in the Exclusion domain (F5), where after accounting for reverse coding, 91% did not feel disadvantaged due to their cultural background or sexuality/gender, suggesting that the sensation of exclusion is not widely experienced among the participants. The Health literacy domain (F9) exhibited a high level of agreement at 87%, indicating that respondents are generally confident in their understanding of health-related information.

The factors of the 11 dimensions structure are sufficiently distinct (see Table 3). The correlations between the factors ranged from 0.074 (Exclusion and Political representation) to 0.631 (Relaxation and Enjoyment). In addition to the support for the discriminant validity, the interrelatedness amongst individual items within a factor was sufficient. The Cronbach’s alpha’s of the factors ranged from 0.71 (Perceived environmental safety) to 0.89 (Political representation). The covariance table (see Supplemental Table 5) also suggests that there are significant and positive interrelations among the latent constructs, indicating a robust fit of the theoretical model to the data.

**Table 3:** Correlations among the factors based on the test dataset

	M	SD	F1	F2	F3	F4	F5	F6	F7	F8	F9	F10	F11
F1	3.67	0.78	(0.86)										
F2	4.05	0.68	0.402***	(0.80)									
F3	3.71	0.83	0.389***	0.612***	(0.84)								
F4	4.02	0.58	0.402***	0.346***	0.223***	(0.71)							
F5	4.36	0.65	0.137**	0.223***	0.145**	0.282***	(0.87)						
F6	3.88	0.63	0.456***	0.466***	0.320***	0.438***	0.223***	(0.83)					
F7	3.92	0.76	0.368***	0.495***	0.378***	0.332***	0.274***	0.368***	(0.88)				
F8	2.91	0.92	0.169***	0.265***	0.205***	0.256***	0.074	0.255***	0.283***	(0.89)			
F9	4.09	0.52	0.195***	0.378***	0.196***	0.234***	0.330***	0.288***	0.317***	0.207***	(0.74)		
F10	3.23	0.80	0.537***	0.349***	0.290***	0.273***	0.184***	0.352***	0.275***	0.185***	0.175***	(0.78)	
F11	3.98	0.68	0.631***	0.572***	0.485***	0.438***	0.207***	0.550***	0.349***	0.162***	0.281***	0.438***	(0.86)

*Note. *N = 501; F1 = Relaxation, F2 = Autonomy, F3 = Fitness, F4 = Perceived environmental safety, F5 = Exclusion (Reversely coded), F6 = Social support, F7 = Financial resources, F8 = Political representation, F9 = Health literacy, F10 = Resilience, F11 = Enjoyment

The standardized lambda coefficients and corresponding R² values are summarized in Supplemental Table 6. The standardized lambda coefficients and corresponding R² values indicate the strength and explanatory power of each indicator within the model. The lambda coefficients, which range from 0.54 to 0.90, reflect the robustness of the relationship between each observed indicator and its respective latent construct. These coefficients suggest that the majority of the indicators have a strong positive loading on their respective factors, with ‘I am able to enjoy life.’ (λ = 0.89) and ‘I feel confident in the way that politicians handle issues that are important to me.’ (λ = 0.90) showing the highest loadings, indicating particularly strong associations with their respective latent constructs. The R² values, which denote the proportion of variance in the indicators explained by the latent factors, range from 0.34 to 0.88, highlighting that a substantial proportion of the variance in most indicators can be accounted for by the model. For instance, ‘I can afford to eat healthily and participate in physical activities.’ has an R² value of 0.88, suggesting that the latent construct explains 88% of the variance in this indicator.

To examine the concurrent validity, the relationships between the 11-factor model and each of the validation scales were tested (Table 4). We hypothesized that each dimension of the CPHQ must demonstrate a significant relationship with at least one of the validation scales (H1). Demonstrating this is crucial to establish that all dimensions are statistically relevant for commonly used health measurements. Furthermore, we expected significant multiple correlation coefficients (R² values) for the multivariate relationship between the CPHQ and the validation scales (H2). This expectation was based on our anticipation that the CPHQ contributes to health measurement, a function also served by the validation scales. As stated in the analysis section, very high R² values could suggest statistical redundancy between the CPHQ and existing measures.

**Table 4:** Multivariate regression analyses between the 11 factors and validation scales

Scale	Domain	Standardized loadings (β) on Positive Health dimensions												
		F1	F2	F3	F4	F5	F6	F7	F8	F9	F10	F11		R^2^
BRS	Resilience	0.195***	0.062	0.018	-0.002	0.011	0.065	0.008	0.065*	0.034	0.755***	0.077		0.567
HR-SWB	Physical independence	0.006	0.173**	0.402***	0.062	0.028	-0.102*	0.028	-0.059	-0.005	-0.029	0.007		0.262
	Happiness	0.081**	-0.019	-0.015	-0.003	-0.022	0.063**	0.020	-0.005	-0.064**	-0.006	0.858***		0.834
	Loneliness	0.297***	-0.025	-0.033	0.021	0.114***	0.251***	0.073	-0.054	-0.012	-0.065	0.177**		0.427
	Autonomy	0.206***	0.166**	-0.122*	0.037	0.015	0.108*	0.044	-0.043	0.158**	0.010	0.024		0.194
	Personal growth	-0.207***	0.078	0.073	-0.011	-0.003	0.001	-0.128*	0.082	0.205***	0.023	0.249***		0.118
EQ-5D	Mobility	-0.001	0.232***	0.563***	-0.092*	0.08*	-0.058	-0.038	0.056	-0.075	-0.013	-0.129*		0.392
	Self-care	-0.080	0.203**	0.183**	-0.017	0.085	-0.076	-0.020	0.010	0.005	0.034	0.106		0.115
	Usual activities	0.017	0.385***	0.379***	-0.066	-0.077*	-0.069	0.108*	0.025	-0.058	-0.037	-0.074		0.416
	Pain/Discomfort	0.106*	0.130*	0.525***	-0.100*	-0.017	-0.045	0.085	0.061	-0.055	-0.025	-0.125*		0.361
	Anxiety/Depression	0.397***	0.013	-0.009	-0.070	-0.129***	0.186***	0.064	0.005	-0.082*	-0.175***	0.216***		0.489
	EQ-VAS	0.152**	0.122*	0.409***	-0.030	-0.010	-0.008	0.071	0.006	-0.020	-0.033	0.118*		0.446
ICECAP-A	Stability	0.278***	0.131*	-0.036	0.081	-0.081	0.085	0.008	0.035	0.002	-0.072	0.061		0.234
	Attachment	0.065	0.021	-0.047	0.049	0.038	0.250***	-0.065	0.055	-0.058	-0.002	0.328***		0.293
	Autonomy	0.125*	0.296***	0.083	0.015	-0.034	0.061	0.021	-0.016	0.029	-0.075	-0.028		0.208
	Achievement	0.097	0.347***	0.111*	-0.009	-0.116**	0.074	0.016	0.005	-0.011	0.020	0.123*		0.332
	Enjoyment	0.224***	0.169**	-0.005	0.032	-0.097**	0.180***	-0.044	0.037	-0.056	0.026	0.300***		0.441

Note. *N* = 501; *R*^*2*^ denotes the amount of variance explained by the six domains of Positive Health; F1 = Relaxation, F2 = Autonomy, F3 = Fitness, F4 = Perceived environmental safety, F5 = Exclusion (Reversely coded), F6 = Social support, F7 = Financial resources, F8 = Political representation, F9 = Health literacy, F10 = Resilience, F11 = Enjoyment; *BRS* = Brief Resilience Scale; *HR-SWB* = Health-Related Subjective Well-Being; *EQ-5D* = EuroQol 5-Dimensional; *EQ-VAS* = EuroQol Visual Analog Scale; *ICECAP-A* = ICEpop CAPability measure for Adults

* = *p* < 0.05

** = *p* < 0.01

*** = *p* < 0.001

Testing the multiple correlation coefficient (i.e., R^2^) between the 11-factor model and the validation scales (H1), we found relationships of mixed strength. The 11-factor model explained less than 25% of the variance of some of the validation scales: *self-care* (EQ-5D, R^2^ = 0.12), *personal growth* (HR-SWB, *R*^2^ = 0.12), *autonomy* (HR-SWB, *R*^2^ = 0.19; ICECAP-A, *R*^2^ = 0.17), and *stability* (ICECAP-A, *R*^2^ = 0.23). Between 25% and 40% was explained by the model in validation scales: *physical independence* (HR-SWB, *R*^*2*^ = 0.26), *attachment* (ICECAP-A, *R*^2^ = 0.29), *achievement* (ICECAP-A, *R*^2^ = 0.33), *pain/discomfort* (EQ-5D, *R*^2^ = 0.36), and *mobility* (EQ-5D, *R*^2^ = 0.39). The model explained more than 40% of the variance in validation scales: *usual activities* (EQ-5D, *R*^2^ = 0.42), *loneliness* (HR-SWB, *R*^2^ = 0.43), *enjoyment* (ICECAP-A, *R*^2^ = 0.44), *EQ-VAS* (EQ-5D, *R*^2^ = 0.45), *anxiety/depression* (EQ-5D, *R*^2^ = 0.49), *resilience* (BRS, *R*^2^ = 0.57), and *happiness* (HR-SWB, *R*^2^ = 0.83). These findings support Hypothesis 1, which posited significant multiple correlation coefficients (R² values) for the multivariate relationship between the CPHQ and the validation scales. While some dimensions, such as happiness (HR-SWB, R² = 0.83) and resilience (BRS, R² = 0.57), demonstrated strong relationships, indicating a substantial overlap with the CPHQ model, others, like self-care (EQ-5D, R² = 0.12), exhibited weaker associations. This variation highlights the multifaceted nature of the CPHQ dimensions and their differential impact on health outcomes, as measured by the validation scales.

Testing the relationship between the 11 factors and the validation scales (H2), we found that *all* factors were statistically important in explaining variance across the validation scales (Table 4). However, five factors (i.e., F1, Autonomy, Fitness, Resilience, Enjoyment) were well covered by the validation scales. For example, *F1* had a strong statistical significant association with *anxiety/depression* (EQ-5D, β = 0.397, *p* < 0.001); *Autonomy* was an important predictor of *usual activities* (EQ-5D, β = 0.385, *p* < 0.001) and *achievement* (ICECAP-A, β = 0.347, *p* < 0.001); *Fitness* was strongly related to among others *mobility* (EQ-5D, β = 0. 563, *p* < 0.001), *pain/discomfort* (EQ-5D, β = 0.563, *p* < 0.001), and *physical independence* (HR-SWB, β = 0. 402, *p* < 0.001); *Resilience* had also a strong statistical relationship with the *Brief Resilience Scale* (BRS, β = 0.755, *p* < 0.001); and *Enjoyment* was an important predictor of *happiness* (HR-SWB, β = 0.858, *p* < 0.001). The remaining six factors (i.e., Perceived environmental safety to Health literacy) were statistically important in explaining variance in the validation scales but less clearly related to one of the validation scales. In line with Hypothesis 2, these findings indicate the statistical relevance of each CPHQ dimension with at least one validation scale, thereby suggesting that all dimensions are statistically significant for commonly used health measurements.

## Discussion

This study aimed to develop a context-sensitive Positive Health Questionnaire (CPHQ), extending previous efforts to develop a Positive Health (PH) measurement model. Compared to the PH measurement model, the CPHQ includes context items following the constitutive elements of the Capability Approach.(14 see Supplemental Table 1). The Capability Approach served as a theoretical framework, responding to calls for a theoretical framework to build and test theory [6, 7], clarity about the focus of CPHQ (on the “ability to adapt”), accounting for health inequality [11], and more comprehensible measurement items [7]. Input from citizens and professionals on Positive Health and context items was included in the development of CPHQ to account for content validity. Factor analysis and regression analysis were conducted to assess the factorial validity and concurrent validity.

An initial questionnaire, which was developed based on PH and the Capability Approach, was refined during focus discussions and expert consultation. The refined questionnaire contained items related to Positive Health and factors related to resilience, social support, relaxation, and autonomy. This refined questionnaire was used during a factor analysis, for which data were gathered among a representative panel of Dutch citizens. The Exploratory Factor Analysis (EFA) suggested a model containing 11 dimensions, which we labeled as relaxation, autonomy, fitness, perceived environmental safety, exclusion, social support, financial resources, political representation, health literacy, resilience, and enjoyment. The factors partly overlap with the initial PH questionnaire, which contains dimensions: physical fitness, mental functions, future perspective, contentment, social relations, and daily life-management [8]. Therefore, the CPHQ advances the PH measurement model, responding to the call from our focus group participants and expert consultation. The integration of the Capability Approach responds to the call for a theoretical framework for PH [6, 7]. The factorial validity of the 11-dimensional CPHQ was supported by Confirmatory Factor Analysis (CFA).

The tests of concurrent validity showed that all 11 dimensions of the CPHQ were statistically important in explaining variance across the validation scales. Five factors were well covered by the validation scales. In particular, these factors showed a strong relationship with *Anxiety/Depression*, *Achievement*, *Mobility*, *Pain/Discomfort*, *Physical independence*, *Resilience*, and *Happiness*. The remaining factors had significant but weak relationships with the validation scales. The factors that showed weaker relationships with the validation scales were mostly the newly added items on context (i.e., perceived environmental safety, social support, exclusion, financial resources, health literacy, and political representation), which were formulated in line with the principles of the Capability Approach [14]. Possibly, these factors affect the extent to which persons can feel well, as we will discuss next.

Applying the Capability Approach as a framework for the CPHQ helps to focus on how health, as a functioning, can be achieved by analyzing a person’s resources as well as conversion factors that could influence the transformation of such resources into health capabilities. For example, *Perceived environmental safety* was characterized by contextual items related to connectedness and feeling safe in one’s environment and was significantly related to the *Mobility* and the *Pain/Discomfort* domains of the EQ-5D. A safe environment is a conversion factor that impacts the ability to control one’s life (capability) as well as impacting actual behavior such as physical activity (functionings), which may explain the specific link between mobility and pain [26]. However, as we did not find a strong correlation with other validation scales, this factor may represent a distinct environmental stressor related to perception instead of actual exposure and act as a personal conversion factor that impacts mobility decisions (e.g., a chosen mode of transportation), for example, due to noise and traffic pollution and dissatisfaction related to relaxation [27]. *Exclusion*, on the other hand, was significantly but weakly related to *Anxiety/Depression*, *Achievements*, and *Loneliness*, which was in the expected direction as discrimination has been found to affect mental health [28] as well as human capital [29]. In this situation, the domain *Exclusion* can act as a conversion factor, whereas the domain *Social Support*, which was related to the items *Loneliness* (HR-SWB), *Attachment* (ICECAP-A), and *Anxiety/Depression* (EQ-5D), included items such as “feeling supported if needed” and “ability to find people to engage with,” which combines personal conversion factor and capabilities.

Similarly, the factor *Financial resources* was characterized by having sufficient financial resources to live a healthy life and had a weak statistical significant relationship with *Personal growth* (HR-SWB) and *Usual activities* (EQ-5D). Financial hardship occurs when one has insufficient financial resources to adequately meet a household’s needs. Experiencing this type of deprivation can impact health and well-being by inducing psychological distress, lack of access to health-promoting resources such as sports and healthy food as well as little participation in leisure activities [30]. 

At last, both *Health literacy* and *Political representation* did not show a strong relationship with the validation scales, which may be explained by the fact that none of the validation scales adequately captured these domains. However, in light of the Capability Approach, both *Health literacy* and *Political representation* are relevant to consider when measuring health. For example, previous work by Pithara [31] has re-conceptualized health literacy by using the Capability Approach in which the authors highlight the need for addressing health literacy as a capability alongside other health-promoting factors instead of focusing on narrow competency-related goals that are mostly used in health literacy measurement scales [32]. Indeed, the current CPHQ represents a combination of health literacy factors combining the ability to communicate with health professionals as well as knowing where to go for medical support and understanding medical information. Also, the importance of political representation has been acknowledged in the Capability Approach of human well-being as political representation is an essential conversion factor for access to health-promoting resources [33]. 

Overall, the CPHQ measurement model showed a higher explained variance in resilience and mental health indicators (i.e., Happiness and Anxiety/Depression) and was similar in explaining the other validation scales when compared to the previous PH scale [6]. Possibly, the CPHQ may be better to measure dimensions of health beyond the initial PH scale, taking into account the context of persons as conversion factors.

### Methodological considerations

A strength of this study is the combination of both a data-driven and citizen-driven approach in a representative Dutch sample and its embeddedness in a theoretical framework of the Capability Approach. Previous tests of validity were primarily empirically oriented [6, 7]. However, as highlighted by Borsboom and colleagues, “validity cannot be solved by psychometric techniques or models alone.” [34]. It is necessary to integrate multiple approaches from psychometrics, philosophy, and psychological theory to examine whether the measurements measure what they purport to measure. In our paper, we expanded our tests of validity by combining psychological theory, item construction with participants and experts, as well as comprehensive data analysis.

This study has some limitations. Different from the initial PH questionnaire, not all items of the CPHQ were formulated positively because we identified in the focus groups that vulnerable groups did not recognize themselves in items that were worded too positively. Inclusion of both positive and negative worded items has been suggested to reduce acquiescent response bias [35] but others have shown the opposite [36]. Although we did not find any indication that the reformulation affected the factorial structure and internal validity relative to the initial PH scale, it may have impacted our results related to the BRS as only the positive items of the BRS were included in the analyses following previous work by van Vliet et al. [8]. Further research is necessary to evaluate the impact of positive and negative worded items in the CPHQ and to what extent the factorial structure and internal validity is also applicable in other settings.

Further, we conducted factor analysis with many newly added items, including those related to context. Some of the initial items from the PH model [8] did not remain in the final CPHQ questionnaire. For example, items of the “spiritual/existential domain” and “mental well-being” were not included in the CPHQ. This is most likely explained by the strong relationship between other factors such as enjoyment/contentment and the spiritual/existential domain [6] and anxiety/depression (EQ-5D) and the CPHQ domains of relaxation, exclusion, social support, political representation, and health literacy. Thus, some items of the initial PH model appear to be replaced by related items.

At last, while the CPHQ explained more variance in resilience than the initial PH scale, it still explained a low variation in *autonomy* (ICECAP-A) and *self-care* (EQ-5D), which are factors important to the initial definition of Huber (i.e., “Health as the ability to self-manage”). This may be explained by the narrow scope of the measurements of both constructs. *Autonomy* was measured as “being able to be independent” (ICECAP-A), and *self-care* (EQ-5D) was measured as “being able to wash or clothe.” However, *autonomy* in the context of health is defined as the right of people to make informed decisions about their medical care [37], and *self-care* is often defined as the tasks performed at home by healthy people to prevent illness [38]. These aspects were not included in the validation scales. Future research should aim to expand the scope of the measurements to include a more comprehensive assessment of autonomy and self-care, in line with the initial definition of Huber [4, 5]. 

### Implications and future directions

In light of the previous work of Prinsen and Terwee [7] and Huber et al. [5], we have shown that the initial PH measurement scale developed by Van Vliet et al. [8] can be further advanced by incorporating personal, social, and environmental context items derived from stakeholders and using the Capability Approach as a theoretical framework. The PH dialogue tool is very broad and includes aspects that either reflect health or influence health. This binary focus does not align well with the current paradigm in healthcare and health policy, which often focuses on traditional (disease) endpoints (i.e., “outcomes” in the field of epidemiology) and quality of life measurements.

The PH built on the Capability Approach focuses less on traditional endpoints but more on people’s opportunities and capabilities towards such endpoints. Hence, by applying the Capability Approach, our measurement can help to develop and evaluate policies and other interventions according to their impact on people’s capabilities and not only on their valued health outcomes (actual functionings). Our measurement focuses on the extent to which people are able tohealthy and to what degree they have the resources (e.g., the availability of healthy foods) needed for this capability, and to what extent conversion factors (e.g., living in a food desert) help transform these resources into opportunities to be well. Thus, our approach differs from the concept of PH, defined as “the ability to adapt and self-manage in the face of social, physical, and emotional challenges” [5]. For that reason, we propose a refined definition:

*“The extent to which one is capable to adapt and to thrive given one’s physical, mental, social and contextual opportunities”*.

Further efforts are needed to test the reproducibility of the CPHQ, the responsiveness to change (i.e., to interventions), and the predictive validity (i.e., to biomedical indicators and healthcare utilization) to further test the construct validity and to create support for the use of CPHQ as a measurement scale in healthcare and policy making.

## Conclusion

This study aimed to further develop and test a context-sensitive measurement of PH (CPHQ). By using a multimethodological approach, we advanced the initial PH questionnaire and added contextual items following the constitutive elements of the Capability Approach. The developed CPHQ showed adequate factorial validity and concurrent validity. Moreover, it explicitly accounts for resilience, which is a central element of the concept of PH. Further research is necessary to establish the relevance of self-management in de CPHQ and to assess its reproducibility, responsiveness, and predictive validity.

### Electronic supplementary material

Below is the link to the electronic supplementary material.

Supplementary Material 1

Supplementary Material 2

## Data Availability

The datasets used and/or analyzed during the current study are available from the corresponding author on reasonable request.

## Abbreviations

BRS: Brief Resilience Scale
CA: Capability Approach
CFA: Confirmatory Factor Analysis
CFI: Comparative Fit Index
Context: sensitive Positive Health Questionnaire
CPHQ: Context sensitive Positive Health Questionnaire
EFA: Exploratory Factor Analysis
EQ-5D: EuroQol five-Dimensions
FA: Factor Analysis
HR-SWB: Health-Related Subjective Well-Being
ICECAP-A: ICEpop CAPability measure for Adults
KMO: Kaiser-Meyer-Olkin
ML: Maximum Likelihood
MLR: robust Maximum Likelihood
PH: Positive Health
RMSA: Root Mean Square Error of Approximation
SRMR: Standardized Root Mean Square Residual
TLI: Tucker-Lewis Index
