# Supplementary material for: Development and validation of a Context-sensitive Positive Health Questionnaire (CPHQ): A factor analysis and multivariate regression study
Source: J Patient Rep Outcomes. 2024 Apr 12;8:44. doi: 10.1186/s41687-024-00718-8 (PMC11014831; doi:10.1186/s41687-024-00718-8)
Supplement: Supplementary file 2 — Supplementary Material 2 [file 41687_2024_718_MOESM2_ESM.docx]

**Supplementary material**

**Supplemental Table 1:** Central capabilities & Capability approach model, adapted from Chiappero-Martinetti & Venkatapuram, 2014

1. Life
2. Bodily Health.
3. Bodily Integrity.
4. Senses, Imagination, and Thought
5. Emotions
6. Practical Reason.
7. Affiliation (living with or towards others, having social base).
8. Other Species.
9. Play.
10. Control over one's Environment from a political and material perspective

**Supplemental Table 2**: Model fit and variance explained for a series of exploratory factor analyses.

| **Number of factors** | **CFI** | **TLI** | **RMSEA** | **SRMR** | **Eigenvalue of the added factor** | **Total variance explained by the added factor (%)** | **Cumulative variance explained (%)** |
| --- | --- | --- | --- | --- | --- | --- | --- |
| 1 | 1.000 | 1.000 | 0.000 | 0.000 | 31.547 | 28.17 | 28.17 |
| 2 | 1.000 | 1.000 | 0.000 | 0.028 | 5.908 | 5.28 | 33.44 |
| 3 | 0.981 | 0.971 | 0.056 | 0.049 | 4.761 | 4.25 | 37.69 |
| 4 | 0.973 | 0.963 | 0.052 | 0.044 | 3.669 | 3.28 | 40.97 |
| 5 | 0.955 | 0.942 | 0.059 | 0.053 | 3.024 | 2.70 | 43.67 |
| 6 | 0.959 | 0.948 | 0.054 | 0.052 | 2.275 | 2.03 | 45.70 |
| 7 | 0.946 | 0.932 | 0.057 | 0.053 | 2.115 | 1.89 | 47.59 |
| 8 | 0.962 | 0.952 | 0.047 | 0.046 | 1.966 | 1.75 | 49.34 |
| 9 | 0.935 | 0.920 | 0.057 | 0.059 | 1.736 | 1.55 | 50.89 |
| 10 | 0.936 | 0.922 | 0.054 | 0.057 | 1.660 | 1.48 | 52.37 |
| 11 | 0.944 | 0.932 | 0.049 | 0.050 | 1.594 | 1.42 | 53.80 |
| 12 | 0.945 | 0.933 | 0.048 | 0.051 | 1.460 | 1.30 | 55.10 |
| 13 | 0.925 | 0.910 | 0.054 | 0.054 | 1.371 | 1.22 | 56.32 |
| 14 | 0.933 | 0.917 | 0.050 | 0.051 | 1.307 | 1.17 | 57.49 |
| 15 | 0.928 | 0.912 | 0.050 | 0.049 | 1.296 | 1.16 | 58.65 |

*Note.* CFI = Comparative Fit Index; TLI = Tucker-Lewis Index; RMSEA = Root Mean Square Error of Approximation; SRMR = Standardized Root Mean Square Residual

**Supplemental Table 3:** Item-level descriptive statistics and frequency distributions of responses for the 5-point Likert scale

| **Latent Factor** | **Description** | **Strongly disagree** | **Disagree** | **Neither disagree nor agree** | **Agree** | **Strongly agree** |
| --- | --- | --- | --- | --- | --- | --- |
| F1 | I am able to relax when necessary. | 1.80% | 8.78% | 21.76% | 55.29% | 12.38% |
| F1 | I have enough peace of mind. | 4.19% | 14.17% | 26.15% | 45.31% | 10.18% |
| F1 | I am able to unwind. | 1.20% | 3.39% | 18.36% | 59.08% | 17.96% |
| F2 | I am capable of carrying out tasks and activities adequately. | 0.20% | 1.60% | 8.78% | 62.48% | 26.95% |
| F2 | I am able to participate in activities that I value in my daily life (work, study, etc.). | 0.80% | 3.79% | 11.58% | 56.89% | 26.95% |
| F2 | I can work/volunteer. | 2.99% | 6.39% | 10.18% | 52.30% | 28.14% |
| F3 | I feel healthy. | 1.40% | 7.98% | 18.56% | 55.29% | 16.77% |
| F3 | I feel in good health. | 2.20% | 14.17% | 24.15% | 47.70% | 11.78% |
| F3 | I can move easily, such as climbing stairs, walking, or cycling. | 3.39% | 10.58% | 13.97% | 45.31% | 26.75% |
| F4 | I feel safe in the neighborhood where I now live. | 0.80% | 1.20% | 5.59% | 62.08% | 30.34% |
| F4 | My home environment is safe and provides numerous opportunities to engage in daily life. | 0.20% | 1.60% | 10.58% | 66.07% | 21.56% |
| F4 | I feel connected to the environment where I now live. | 1.40% | 7.58% | 20.76% | 51.70% | 18.56% |
| F5 | I feel disadvantaged because of my religion or spiritual beliefs. | 49.10% | 43.11% | 5.19% | 2.20% | 0.40% |
| F5 | I feel disadvantaged because of my (cultural) background. | 47.90% | 43.91% | 5.79% | 2.20% | 0.20% |
| F5 | I feel disadvantaged or excluded based on my sexuality and/or gender. | 46.31% | 42.91% | 7.58% | 2.79% | 0.40% |
| F6 | I can find people with whom I can have a good time. | 1.00% | 3.79% | 19.56% | 61.68% | 13.97% |
| F6 | I feel that people support me when needed. | 0.40% | 2.40% | 15.17% | 65.07% | 16.97% |
| F6 | I feel that I 'fit in' in my environment. | 1.40% | 4.19% | 17.37% | 63.87% | 13.17% |
| F7 | I can afford to eat healthily and participate in physical activities. | 1.00% | 4.79% | 13.77% | 57.88% | 22.55% |
| F7 | I have enough money to do things that are important to me. | 2.79% | 9.38% | 15.57% | 53.49% | 18.76% |
| F7 | I can afford to live a healthy lifestyle. | 0.80% | 3.39% | 13.17% | 55.29% | 27.35% |
| F8 | Politics makes me feel represented. | 10.58% | 23.95% | 41.92% | 20.76% | 2.79% |
| F8 | I feel confident in the way that politicians handle issues that are important to me. | 8.38% | 17.76% | 42.51% | 27.35% | 3.99% |
| F9 | I can communicate with healthcare professionals and understand their explanations of my illness or treatment. | 0.20% | 0.60% | 12.97% | 60.08% | 26.15% |
| F9 | I know where to go for medical assistance. | 0.20% | 0.80% | 6.19% | 63.27% | 29.54% |
| F9 | When I look up or receive information about a subject, it is explained in a way that I can understand. | 0.40% | 2.00% | 16.97% | 65.07% | 15.57% |
| F10 | When something bad happens, it is difficult for me to move on. | 4.39% | 17.96% | 32.73% | 40.12% | 4.79% |
| F10 | I have a hard time getting through stressful situations. | 5.39% | 25.15% | 30.54% | 33.13% | 5.79% |
| F10 | I don't need much time to recover from a stressful event. | 3.39% | 13.57% | 31.94% | 45.11% | 5.99% |
| F11 | I feel happy. | 1.60% | 5.39% | 18.56% | 57.09% | 17.37% |
| F11 | I am able to enjoy life. | 1.00% | 2.79% | 14.17% | 59.28% | 22.75% |
| F11 | I am able to be grateful for what life has to offer. | 0.60% | 2.59% | 9.78% | 60.88% | 26.15% |

*Note.* F1 = Relaxation, F2 = Autonomy, F3 = Fitness, F4 = Perceived environmental safety, F5 = Exclusion, F6 = Social support, F7 = Financial resources, F8 = Political representation, F9 = Health literacy, F10 = Resilience, F11 = Enjoyment.

**Supplemental Table 4:** Demographic characteristics of the survey panel and comparison with the general Dutch Population.

| **Gender** | **Respondents** | **CBS 2020** |
| --- | --- | --- |
| Male | 50% | 49% |
| Female | 50% | 51% |
| Total | 100% | 100% |

| **Age** | **Responses** | **CBS 2020** |
| --- | --- | --- |
| 18–19 years | 1% | 3% |
| 20–24 years | 5% | 8% |
| 25–29 years | 8% | 8% |
| 30–34 years | 7% | 8% |
| 35–39 years | 7% | 8% |
| 40–44 years | 7% | 7% |
| 45–49 years | 9% | 9% |
| 50–54 years | 10% | 9% |
| 55–59 years | 10% | 9% |
| 60–64 years | 9% | 8% |
| 65 years and older | 28% | 23% |
| Total | 100% | 100% |

| **Educational Level** | **Responses** | **CBS 2020** |
| --- | --- | --- |
| Low | 28% | 28% |
| Medium | 44% | 42% |
| High | 28% | 30% |
| Total | 100% | 100% |

| **Province** | **Responses** | **CBS 2020** |
| --- | --- | --- |
| Drenthe | 3% | 3% |
| Flevoland | 3% | 2% |
| Friesland | 4% | 4% |
| Gelderland | 12% | 12% |
| Groningen | 2% | 3% |
| Limburg | 8% | 7% |
| North Brabant | 15% | 15% |
| North Holland | 18% | 17% |
| Overijssel | 6% | 7% |
| Utrecht | 7% | 8% |
| Zeeland | 3% | 2% |
| South Holland | 20% | 21% |
| Total | 100% | 100% |

*Note.* CBS = Statistics Netherlands

**Supplemental Table 5:** Estimated covariance matrix from Confirmatory Factor Analysis (CFA)

| **Latent Factor 1** | **Latent Factor 2** | **Covariance** | **Standardized Covariance** |
| --- | --- | --- | --- |
| I feel happy. | I feel happy. | 0.156 | 0.226 |
| I am able to enjoy life. | I am able to enjoy life. | 0.120 | 0.211 |
| I am able to be grateful for what life has to offer. | I am able to be grateful for what life has to offer. | 0.262 | 0.515 |
| I am able to relax when necessary. | I am able to relax when necessary. | 0.150 | 0.200 |
| I have enough peace of mind. | I have enough peace of mind. | 0.328 | 0.333 |
| I am able to unwind. | I am able to unwind. | 0.226 | 0.379 |
| I am capable of carrying out tasks and activities adequately. | I am capable of carrying out tasks and activities adequately. | 0.161 | 0.384 |
| I am able to participate in activities that I value in my daily life (work, study, etc.). | I am able to participate in activities that I value in my daily life (work, study, etc.). | 0.135 | 0.223 |
| I can work/volunteer. | I can work/volunteer. | 0.476 | 0.525 |
| I feel healthy. | I feel healthy. | 0.156 | 0.208 |
| I feel in good health. | I feel in good health. | 0.214 | 0.237 |
| I can move easily, such as climbing stairs, walking, or cycling. | I can move easily, such as climbing stairs, walking, or cycling. | 0.621 | 0.564 |
| When something bad happens, it is difficult for me to move on. | When something bad happens, it is difficult for me to move on. | 0.280 | 0.313 |
| I have a hard time getting through stressful situations. | I have a hard time getting through stressful situations. | 0.397 | 0.389 |
| I don't need much time to recover from a stressful event. | I don't need much time to recover from a stressful event. | 0.515 | 0.623 |
| I can find people with whom I can have a good time. | I can find people with whom I can have a good time. | 0.238 | 0.433 |
| I feel that people support me when needed. | I feel that people support me when needed. | 0.161 | 0.356 |
| I feel that I 'fit in' in my environment. | I feel that I 'fit in' in my environment. | 0.192 | 0.337 |
| I feel safe in the neighborhood where I now live. | I feel safe in the neighborhood where I now live. | 0.126 | 0.286 |
| My home environment is safe and provides numerous opportunities to engage in daily life. | My home environment is safe and provides numerous opportunities to engage in daily life. | 0.233 | 0.585 |
| I feel connected to the environment where I now live. | I feel connected to the environment where I now live. | 0.513 | 0.661 |
| I feel disadvantaged because of my religion or spiritual beliefs. | I feel disadvantaged because of my religion or spiritual beliefs. | 0.127 | 0.243 |
| I feel disadvantaged because of my (cultural) background. | I feel disadvantaged because of my (cultural) background. | 0.105 | 0.208 |
| I feel disadvantaged or excluded based on my sexuality and/or gender. | I feel disadvantaged or excluded based on my sexuality and/or gender. | 0.266 | 0.455 |
| I can afford to eat healthily and participate in physical activities. | I can afford to eat healthily and participate in physical activities. | 0.144 | 0.223 |
| I have enough money to do things that are important to me. | I have enough money to do things that are important to me. | 0.247 | 0.270 |
| I can afford to live a healthy lifestyle. | I can afford to live a healthy lifestyle. | 0.215 | 0.352 |
| I can communicate with healthcare professionals and understand their explanations of my illness or treatment. | I can communicate with healthcare professionals and understand their explanations of my illness or treatment. | 0.143 | 0.340 |
| I know where to go for medical assistance. | I know where to go for medical assistance. | 0.144 | 0.399 |
| When I look up or receive information about a subject, it is explained in a way that I can understand. | When I look up or receive information about a subject, it is explained in a way that I can understand. | 0.308 | 0.705 |
| Politics makes me feel represented. | Politics makes me feel represented. | 0.201 | 0.212 |
| I feel confident in the way that politicians handle issues that are important to me. | I feel confident in the way that politicians handle issues that are important to me. | 0.180 | 0.190 |
| F11 | F11 | 0.535 | 1.000 |
| F1 | F1 | 0.599 | 1.000 |
| F2 | F2 | 0.258 | 1.000 |
| F3 | F3 | 0.594 | 1.000 |
| F10 | F10 | 0.615 | 1.000 |
| F6 | F6 | 0.312 | 1.000 |
| F4 | F4 | 0.314 | 1.000 |
| F5 | F5 | 0.393 | 1.000 |
| F7 | F7 | 0.500 | 1.000 |
| F9 | F9 | 0.277 | 1.000 |
| F8 | F8 | 0.746 | 1.000 |
| F11 | F1 | 0.401 | 0.708 |
| F11 | F2 | 0.252 | 0.680 |
| F11 | F3 | 0.309 | 0.548 |
| F11 | F10 | 0.280 | 0.488 |
| F11 | F6 | 0.267 | 0.654 |
| F11 | F4 | 0.220 | 0.537 |
| F11 | F5 | 0.097 | 0.212 |
| F11 | F7 | 0.200 | 0.387 |
| F11 | F9 | 0.130 | 0.336 |
| F11 | F8 | 0.114 | 0.180 |
| F1 | F2 | 0.200 | 0.509 |
| F1 | F3 | 0.260 | 0.436 |
| F1 | F10 | 0.358 | 0.589 |
| F1 | F6 | 0.231 | 0.534 |
| F1 | F4 | 0.208 | 0.479 |
| F1 | F5 | 0.066 | 0.137 |
| F1 | F7 | 0.221 | 0.404 |
| F1 | F9 | 0.108 | 0.264 |
| F1 | F8 | 0.130 | 0.194 |
| F2 | F3 | 0.250 | 0.639 |
| F2 | F10 | 0.164 | 0.411 |
| F2 | F6 | 0.159 | 0.561 |
| F2 | F4 | 0.129 | 0.454 |
| F2 | F5 | 0.081 | 0.253 |
| F2 | F7 | 0.200 | 0.557 |
| F2 | F9 | 0.126 | 0.473 |
| F2 | F8 | 0.122 | 0.279 |
| F3 | F10 | 0.192 | 0.317 |
| F3 | F6 | 0.152 | 0.353 |
| F3 | F4 | 0.121 | 0.281 |
| F3 | F5 | 0.067 | 0.139 |
| F3 | F7 | 0.230 | 0.423 |
| F3 | F9 | 0.081 | 0.199 |
| F3 | F8 | 0.151 | 0.227 |
| F10 | F6 | 0.177 | 0.404 |
| F10 | F4 | 0.148 | 0.338 |
| F10 | F5 | 0.097 | 0.197 |
| F10 | F7 | 0.165 | 0.297 |
| F10 | F9 | 0.101 | 0.243 |
| F10 | F8 | 0.126 | 0.186 |
| F6 | F4 | 0.162 | 0.516 |
| F6 | F5 | 0.090 | 0.257 |
| F6 | F7 | 0.168 | 0.424 |
| F6 | F9 | 0.107 | 0.362 |
| F6 | F8 | 0.145 | 0.301 |
| F4 | F5 | 0.135 | 0.386 |
| F4 | F7 | 0.170 | 0.429 |
| F4 | F9 | 0.109 | 0.371 |
| F4 | F8 | 0.149 | 0.308 |
| F5 | F7 | 0.130 | 0.294 |
| F5 | F9 | 0.131 | 0.398 |
| F5 | F8 | 0.043 | 0.079 |
| F7 | F9 | 0.145 | 0.391 |
| F7 | F8 | 0.195 | 0.319 |
| F9 | F8 | 0.107 | 0.234 |

*Note.* F1 = Relaxation, F2 = Autonomy, F3 = Fitness, F4 = Perceived environmental safety, F5 = Exclusion, F6 = Social support, F7 = Financial resources, F8 = Political representation, F9 = Health literacy, F10 = Resilience, F11 = Enjoyment.

**Supplemental Table 6:** Standardized lambda (λ) coefficients and R2 values for the 32 indicators

| **Indicator** | **F1** | **F2** | **F3** | **F4** | **F5** | **F6** | **F7** | **F8** | **F9** | **F10** | **F11** | **R2** |
| --- | --- | --- | --- | --- | --- | --- | --- | --- | --- | --- | --- | --- |
| I am able to relax when necessary. | 0.89 |  |  |  |  |  |  |  |  |  |  | 0.80 |
| I have enough peace of mind. | 0.82 |  |  |  |  |  |  |  |  |  |  | 0.67 |
| I am able to unwind. | 0.79 |  |  |  |  |  |  |  |  |  |  | 0.62 |
| I am capable of carrying out tasks and activities adequately. |  | 0.79 |  |  |  |  |  |  |  |  |  | 0.62 |
| I am able to participate in activities that I value in my daily life (work, study, etc.). |  | 0.88 |  |  |  |  |  |  |  |  |  | 0.78 |
| I can work/volunteer. |  | 0.69 |  |  |  |  |  |  |  |  |  | 0.47 |
| I feel healthy. |  |  | 0.89 |  |  |  |  |  |  |  |  | 0.79 |
| I feel in good health. |  |  | 0.87 |  |  |  |  |  |  |  |  | 0.76 |
| I can move easily, such as climbing stairs, walking, or cycling. |  |  | 0.66 |  |  |  |  |  |  |  |  | 0.44 |
| I feel safe in the neighborhood where I now live. |  |  |  | 0.84 |  |  |  |  |  |  |  | 0.71 |
| My home environment is safe and provides numerous opportunities to engage in daily life. |  |  |  | 0.64 |  |  |  |  |  |  |  | 0.41 |
| I feel connected to the environment where I now live. |  |  |  | 0.58 |  |  |  |  |  |  |  | 0.34 |
| I feel disadvantaged because of my religion or spiritual beliefs. |  |  |  |  | 0.87 |  |  |  |  |  |  | 0.76 |
| I feel disadvantaged because of my (cultural) background. |  |  |  |  | 0.89 |  |  |  |  |  |  | 0.79 |
| I feel disadvantaged or excluded based on my sexuality and/or gender. |  |  |  |  | 0.74 |  |  |  |  |  |  | 0.55 |
| I can find people with whom I can have a good time. |  |  |  |  |  | 0.75 |  |  |  |  |  | 0.57 |
| I feel that people support me when needed. |  |  |  |  |  | 0.80 |  |  |  |  |  | 0.64 |
| I feel that I 'fit in' in my environment. |  |  |  |  |  | 0.81 |  |  |  |  |  | 0.66 |
| I can afford to eat healthily and participate in physical activities. |  |  |  |  |  |  | 0.88 |  |  |  |  | 0.88 |
| I have enough money to do things that are important to me. |  |  |  |  |  |  | 0.85 |  |  |  |  | 0.85 |
| I can afford to live a healthy lifestyle. |  |  |  |  |  |  | 0.80 |  |  |  |  | 0.80 |
| Politics makes me feel represented. |  |  |  |  |  |  |  | 0.89 |  |  |  | 0.79 |
| I feel confident in the way that politicians handle issues that are important to me. |  |  |  |  |  |  |  | 0.90 |  |  |  | 0.81 |
| I can communicate with healthcare professionals and understand their explanations of my illness or treatment. |  |  |  |  |  |  |  |  | 0.81 |  |  | 0.81 |
| I know where to go for medical assistance. |  |  |  |  |  |  |  |  | 0.78 |  |  | 0.78 |
| When I look up or receive information about a subject, it is explained in a way that I can understand. |  |  |  |  |  |  |  |  | 0.54 |  |  | 0.54 |
| When something bad happens, it is difficult for me to move on. |  |  |  |  |  |  |  |  |  | 0.83 |  | 0.83 |
| I have a hard time getting through stressful situations. |  |  |  |  |  |  |  |  |  | 0.78 |  | 0.78 |
| I don't need much time to recover from a stressful event. |  |  |  |  |  |  |  |  |  | 0.61 |  | 0.61 |
| I feel happy. |  |  |  |  |  |  |  |  |  |  | 0.88 | 0.77 |
| I am able to enjoy life. |  |  |  |  |  |  |  |  |  |  | 0.89 | 0.79 |
| I am able to be grateful for what life has to offer. |  |  |  |  |  |  |  |  |  |  | 0.70 | 0.48 |

*Note.* F1 = Relaxation, F2 = Autonomy, F3 = Fitness, F4 = Perceived environmental safety, F5 = Exclusion, F6 = Social support, F7 = Financial resources, F8 = Political representation, F9 = Health literacy, F10 = Resilience, F11 = Enjoyment.
