# Supplementary material for: Development and validation of a Context-sensitive Positive Health Questionnaire (CPHQ): A factor analysis and multivariate regression study
Source: J Patient Rep Outcomes. 2024 Apr 12;8:44. doi: 10.1186/s41687-024-00718-8 (PMC11014831; doi:10.1186/s41687-024-00718-8)
Supplement: Supplementary file 1 — Supplementary Material 1 [file 41687_2024_718_MOESM1_ESM.docx]

Dear Dr. Ruseckaite and Reviewers,

Thank you for the opportunity to revise our manuscript titled 'Development and validation of a Context-sensitive Positive Health Questionnaire (CPHQ): A factor analysis and multivariate regression study'. We appreciate the assessment and constructive criticism provided by both reviewers, which has strengthened the quality and clarity of our work.

In response to Reviewer #1's comments, we have taken the suggestion to restructure the manuscript into consideration. We propose renaming the first two phases as the Preparation phase, as it accurately reflects the foundational steps taken in developing the CPHQ. This restructuring will enhance the readability and transparency of our methodology. Additionally, we have clarified the composition of the focus groups and addressed concerns regarding the distribution of item-level data, particularly in the Exclusion domain.

To Reviewer #2, we are grateful for acknowledging the adequacy of our revisions.

Attached is our revised manuscript, incorporating the suggested changes and addressing all points raised by the reviewers. We have also updated our manuscript to include the actual details of our funding and ethics approval, previously anonymized as "XXXX". Once again, we would like to express our gratitude for the opportunity to revise and resubmit our manuscript. We look forward to hearing from you regarding the next steps in the publication process.

With kind regards.

Reviewer #1:

1. *Extensive revisions have been made to the original paper that effectively address the majority of the issues noted in the first review, particularly in relation to the focus groups, expert consultants, and the rationale for the quantitative analyses. There are, however, a few points that require additional clarification.*

*The current organization of the paper makes it difficult to follow the three phases of the research. All information about the focus group and expert consultation phases is subsumed in the Methods section, while the Analyses and Results sections apply only to the quantitative analyses of the survey data. It would improve clarity if there could be a section for each phase with Methods, Analyses and Results in each of the three sections.*

Thank you for your valuable feedback. We agree that restructuring the three phases of our research could enhance the clarity of the manuscript. We propose renaming the first two phases - focus groups and expert consultations - as the Preparation phase. This change more accurately reflects their role as foundational steps in developing the Context-sensitive Positive Health Questionnaire (CPHQ).

The preparation phase, encompassing both focus groups and expert consultations, was instrumental in gathering insights from diverse participant groups and experts. This phase was pivotal in refining the questionnaire, ensuring its relevance and comprehensibility, and was not primarily aimed at collecting quantitative data but at preparing the questionnaire for the subsequent validation phase.

In our revised Methods section, we will clearly outline the activities of the preparation phase, including both focus groups and expert consultations. For a detailed overview of the outcomes from this phase, we direct readers to Supplemental Table 1. We are confident that this restructuring will enhance the manuscript's readability and transparently convey the process we followed in developing the CPHQ.

2. *Some additional information about the composition of the focus groups is needed. It is not clear whether the groups were formed to be homogenous with respect to some of the demographic characteristics that are mentioned or whether all were heterogeneous. Both are acceptable approaches but may yield different results. Therefore, it is important to explain how the groups were formed.*

Thank you for your query regarding the composition of the focus groups. We appreciate the opportunity to further clarify our methodological approach and the rationale behind the formation of our focus groups.

The focus groups were not specifically formed to be homogeneous or heterogeneous with respect to the demographic characteristics we mention in our paper. Instead, we chose a recruitment strategy that aimed to be inclusive of underrepresented populations, particularly those from disadvantaged contexts. Specifically, we recruited participants through general practitioners in areas with socioeconomic challenges. This approach inadvertently resulted in groups that can be considered inherently heterogeneous, reflecting a broad spectrum of societal segments.

To address this aspect, we have updated the methods section of our manuscript to include: "Our recruitment strategy was designed to ensure a diverse and inclusive representation of participants. This included reaching out to potential participants through general practitioners in socioeconomically disadvantaged areas, aiming to capture a wide array of perspectives across different demographic characteristics."

3. *A useful table with item-level information has been added, but this information should be summarized in the Results sections prior to the discussion of the factor analyses. There are several items that seem notably positively skewed. This is not unexpected for some items ("I know where to go for medical assistance," and the last three items, starting with "I feel happy."). However, it is surprising that to see that ~90% of respondents agreed or strongly agreed with the statements, "I feel disadvantaged because of my cultural background," and "I feel disadvantaged or excluded based on my sexuality or gender.") Some interpretation or comment is needed about these answers and why the authors feel these items are performing adequately.*

Thank you for emphasizing the importance of summarizing item-level data in the Results section. In the revised manuscript, we have included a summary that addresses the distribution of the items and notes the skewness observed. We present this summary after establishing that an 11-dimension factor model adequately describes the data, which allows us to focus on dimensions rather than individual items.

We would like to clarify that the items "I feel disadvantaged because of my cultural background" and "I feel disadvantaged or excluded based on my sexuality or gender" are reverse-coded. A higher score indicates a more favorable outcome in the Exclusion domain, meaning that the majority of participants did not report feelings of disadvantage or exclusion. Despite the skewness in the Exclusion items, these are essential for capturing aspects of exclusion, which have a meaningful but weak correlation with factors such as Anxiety/Depression, Achievements, and Loneliness. As mentioned in the paper, these relationships align with literature suggesting that discrimination can adversely affect mental health and human capital. In the revised version of the manuscript, we have corrected our mistake in the item-level table by aligning the percentages with the correct labels - effectively undoing the reverse coding. In the correlation and regression tables, we now indicate that Exclusion was reverse-coded. Thank you for bringing this to our attention.

In the revised manuscript, we state: “Furthermore, to provide a comprehensive understanding of the item-level responses, we have included detailed item descriptives in Supplemental Table 3. This table presents the frequency distribution of responses for each item on the 5-point Likert scale. Notably, in the Autonomy domain (F2), 85% of respondents reported feeling self-sufficient in managing tasks, underscoring a strong perception of individual agency. In the Perceived Environmental Safety domain (F4), 83% of participants agreed they feel secure in their living environments. The Exclusion domain (F5) revealed that, after reverse coding, 91% did not feel disadvantaged due to their cultural background or sexuality/gender, indicating a lower-than-anticipated experience of exclusion among the participants. Additionally, the Health Literacy domain (F9) showed a high level of agreement at 87%, reflecting the respondents' confidence in their understanding of health-related information.”

Reviewer #2:

1. *The authors have addressed adequately my suggestions.*

Thank you sincerely for taking the time to review our manuscript and for your positive feedback on the revisions we made. We are grateful for your valuable input, which has contributed to the improvement of our manuscript and its potential for publication.
